# Effect of a mitochondrial‐targeted coenzyme Q analog on pancreatic β‐cell function and energetics in high fat fed obese mice

**DOI:** 10.1002/prp2.393

**Published:** 2018-06-01

**Authors:** Yumi Imai, Brian D. Fink, Joseph A. Promes, Chaitanya A. Kulkarni, Robert J. Kerns, William I. Sivitz

**Affiliations:** ^1^ Division of Endocrinology and Metabolism Department of Internal Medicine University of Iowa and the Iowa City Veterans Affairs Medical Center Iowa City Iowa USA; ^2^ Department of Pharmaceutical Sciences and Experimental Therapeutics University of Iowa Iowa City Iowa USA

**Keywords:** antioxidants, beta‐cells, coenzyme Q, insulin, mitochondria, obesity

## Abstract

We recently reported that mitoquinone (mitoQ, 500 μmol/L) added to drinking water of C57BL/6J mice attenuated weight gain and reduced oxidative stress when administered to high‐fat (HF) fed mice. Here, we examined the effects of mitoQ administered to HF fed mice on pancreatic islet morphology, dynamics of insulin secretion, and islet mitochondrial metabolism. C57BL/6J mice were fed HF for 130 days while we administered vehicle (cyclodextrin [CD]) or mitoQ added to the drinking water at up to 500 μmol/L. MitoQ‐treated mice vs vehicle gained significantly less weight, expended significantly more energy as determined by indirect calorimetry, and trended to consume less (nonsignificant) food. As we and others reported before, mitoQ‐treated mice drank less water but showed no difference in percent body fluid by nuclear magnetic resonance. Circulating insulin and glucose‐stimulated insulin secretion by isolated islets were decreased in mitoQ‐treated mice while insulin sensitivity (plasma insulin x glucose) was greater. Islet respiration as basal oxygen consumption (OCR), OCR directed at ATP synthesis, and maximal uncoupled OCR were also reduced in mitoQ‐treated mice. Quantitative morphologic studies revealed that islet size was reduced in the mitoQ‐treated mice while visual inspection of histochemically stained sections suggested that mitoQ reduced islet lipid peroxides. MitoQ markedly improved liver function as determined by plasma alanine aminotransferase. In summary, mitoQ treatment reduced the demand for insulin and reduced islet size, likely consequent to the action of mitoQ to mitigate weight gain and improve liver function.

**Abbreviations**
ALT: alanine aminotransferase
BSA: bovine serum albumin
CD: cyclodextrin
CoQ: coenzyme Q
DAB: diaminobenzidine
HF: high fat
MDA: malonylaldehyde
MitoQ: mitoquinone
NMR: nuclear magnetic resonance
OCR: oxygen consumption rate
TPP^+^: tetraphenylphosphonium
VCO_2_: carbon dioxide production
VO_2_: oxygen consumption

## 1 INTRODUCTION

**Figure 1:**
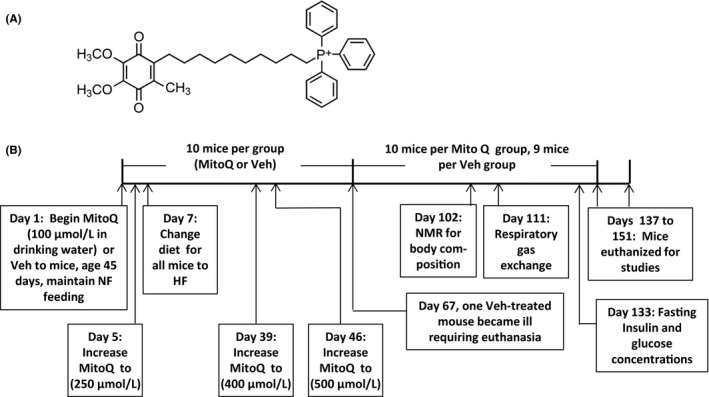
MitoQ structure and study protocol. (A) Structure. (B) Protocol indicating times of treatments and procedures. Veh, vehicle

Mitoquinone (mitoQ, Figure 1A) is a derivative of endogenous coenzyme Q (CoQ) consisting of the quinone moiety and a saturated 10 carbon side chain as opposed to the 50 carbon unsaturated side chain of CoQ.^1^ A cationic moiety, triphenylphosphonium (TPP^+^), is appended to the side chain in order to target mitochondria by virtue of the relative negative charge in the mitochondrial matrix. MitoQ has well‐established antioxidant properties by virtue of its capacity to block lipid peroxidation.^2^ Moreover, evidence from our laboratory and others^3^, ^4^, ^5^, ^6^ have shown that mitoQ or related molecules have metabolic properties as well acting as an uncoupler of mitochondrial oxidative phosphorylation. MitoQ may also suppress appetite and increase insulin sensitivity, suggesting action as an antiobesity and/or antidiabetic agent. Indeed, we and others^7^, ^8^, ^9^ have administered mitoQ to obese or obesity prone mice and found that the compound decreased weight and fat mass and also reduced hepatic oxidative damage due to steatosis.^8^

In contrast with what is known of the action of mitoQ in obesity and insulin resistant states, little is known regarding the effect of mitoQ on pancreatic islet function. The effects of the drug on insulin dynamics when administered to live animals are not clear. We hypothesized that mitoQ would alter pancreatic β‐cell function when administered to high fat (HF)‐fed obesity prone C57BL/6 mice. Here we further examined the effects of mitoQ on body mass and energetics, but with a focus on pancreatic islet parameters including insulin release, islet morphology, and islet oxidative stress.

## 2 MATERIALS AND METHODS

### 2.1 Reagents and supplies

Synthesis of mitoQ mesylate and preparation of the cyclodextrin (CD) complex of mitoQ mesylate are described in detail in supplemental information. All other reagents, kits, and supplies were as specified or purchased from standard sources.

### 2.2 Animal procedures

Male C57BL/6J mice were obtained from Jackson Laboratories (Bar Harbor, ME). Mice were fed a normal rodent diet (13% kcal fat, diet 7001; Teklad, Harlan Labs, Madison, WI) until initiation of the dietary protocol at age 45 days and maintained according to National Institute of Health guidelines. The protocol was approved by our Institutional Animal Care and Use Committee.

The protocol and procedures are depicted in Figure 1B. At 45 days of age (study day 1), mice (n = 10 per group) were randomized with stratification for body mass to treatment with either mitoQ mesylate complexed with CD added to the drinking water or vehicle (Veh) (water plus CD). At study day 5, the concentration of mitoQ in the drinking water was increased from 100 to 250 μmol/L. Two days later (study day 7) all mice were begun on a HF diet (lard, 60% kcal fat, D12492; Research Diets, New Brunswick, NJ) and maintained on this diet until sacrifice at study end. As shown in Figure 1B, mitoQ in the drinking water was adjusted up to 500 μmol/L. Study procedures were performed at the indicated times (Figure 1B).

Within each treatment group, 2 mice were housed per cage. However, one mouse in the vehicle group became ill with dermatitis and weight loss and had to be sacrificed at day 67, so there was one single caged mouse for the remainder of the study. Mice were weighed on study days 0, 2, 4, 7, 11, and then every 7 days. The weight data for the mouse sacrificed on day 67 was excluded from analysis. Water intake (per cage) was measured on days 5, 7, 11, and then every 7 days. Food intake (per cage) was measured on days 7, 11, and then every 7 days. Food and water intake were determined by the difference between the previously added and remaining supplies and expressed per cage divided by the number of mice in each cage (2 for all cages except the above indicated single mouse). The data for food and water intake prior to day 67 for the cage containing the single mouse was excluded for analysis. Mice were euthanized between days 137 and 151 with isoflurane for collection of blood by cardiac puncture and isolation of tissues for study. Mice were allowed to feed overnight before sacrifice in the am at least 2 hours after removal from the cage.

### 2.3 Body composition

Body composition including fat, lean, and fluid mass were determined by nuclear magnetic resonance (NMR) spectroscopy using a Bruker mini spec LF 90II instrument. To analyze body composition mice were placed into a restraint tube and inserted into the rodent‐sized NMR apparatus adjusting the volume of the chamber based on the size of the animal.

### 2.4 Indirect calorimetry (whole animal gas exchange)

A PhysioScan Metabolic System (Omnitech Electronics, Columbus, Ohio USA) was used to assess gas exchange in small animals. Mice were placed within the chamber for 20 minutes. Gas exchange was determined over the last 5 minutes at which time oxygen consumption (VO_2_) had reached a steady plateau in the resting animal. These studies were performed over the course of the afternoon on all mice, staggering the order between vehicle and mitoQ treated animals.

### 2.5 Plasma insulin and glucose and insulin sensitivity

Fasting plasma insulin and glucose were determined on plasma from tail blood at 11 am after withholding food beginning at 5 pm on the previous day. Insulin was determined by ELISA using a kit (EMD Millipore, Burlington, Massachusetts USA) and glucose was assayed with a Contour brand glucose meter and strips (Bayer, Inc. USA). Insulin sensitivity was assessed as the product of glucose and insulin analogous to the homeostasis model (HOMA)^10^ used in humans as insulin x glucose x a constant that does not differ between assays.

### 2.6 Isolation of islets

Pancreatic islets were isolated by collagenase digestion followed by Ficoll density gradient centrifugation.^11^ In brief, pancreatic tissue was digested at 37°C after infusion of collagenase P (Roche, Mannheim, Germany) to the pancreatic duct through the common bile duct clamped at its entrance to duodenum. Then, islets were separated from nonislet tissue by Ficoll density gradient and handpicked under a dissecting microscope for respirometry or perifusion assays.

### 2.7 Islet Respiration and Mitochondrial function

Respirometry of isolated islets (n = 6 mice per group) was performed with an Agilent Seahorse XF24 respirometer (Agilent Technologies, Santa Clara, CA). Islets were transferred to individual wells (80 islets per well) of an islet capture microplate containing respiratory medium consisting of 120 mmol/L NaCl, 4.8 mmol/L KCl, 2.5 mmol/L CaCl2, 1.2 mmol/L MgCl2, 10 mmol/L HEPES pH 7.45, 2.8 mmol/L D‐glucose, and 0.25% defatted bovine serum albumin (BSA). Capture screens were inserted over the islets to prevent islet loss during respirometry. We then sequentially determined the basal oxygen consumption rate (OCR), OCR after increasing the glucose concentration to 16.7 mmol/L, OCR after addition of 5 μmol/L oligomycin to block ATP production, and OCR after addition of 2 μmol/L rotenone and 0.5 μmol/L antimycin A to block all mitochondrial function. The OCR directed at ATP synthesis was calculated as OCR in the presence of glucose minus OCR in the presence of glucose plus oligomycin. Nonmitochondrial OCR was determined as residual respiration after addition of rotenone + antimycin A and subtracted from measured basal OCR and glucose‐stimulated OCR to calculate the actual mitochondrial component.

### 2.8 Basal and glucose‐induced insulin secretion from isolated islets

50 freshly isolated islets were loaded to a perifusion chamber of Biorep perifusion system (Biorep, Miami Lake, FL) and perifused for 48 min with Krebs buffer (pH 7.4) containing 2.5 mmol/L Ca^2+^, 0.25% BSA, 10 mmol/L HEPES, and 2.8 mmol/L glucose at 37°C, followed by Krebs buffer with 16.8 mmol/L glucose for 16 minutes. At the end of each experiment, islets were tested for insulin secretion by adding 30 mmol/L KCl to the perifusate. Samples were collected at 0.1 mL/min for insulin measurement by ELISA (ALPCO, Salem, NH).

### 2.9 Determination of islet size

Prior to placing the islet plates in the respirometer to assess mitochondrial function, islets within the Seahorse islet plates were photographed using a Nikon‐DIAPHOT microscope with 4× phase contrast objective. Image analysis for islet area was performed on a mean of 78 ± 2 islets per plate excluding islets wherein overlap prevented accurate assessment. This was carried out using SIMPLE PCI software version 6.6 by Compix Inc. USA.

### 2.10 Immunochemistry

Tissue sections from 3 mice per group were fixed in 10% formalin, embedded in paraffin, and immunostained for malonylaldehyde (MDA). The slides were deparaffinized and rehydrated through a series of ethanol concentrations. After antigen retrieval with citrate buffer (pH 6.0) at 94°C for 25 minutes, the sections were blocked using normal donkey serum and incubated with 1:500 of goat anti‐MDA antibody (NB100‐62737; Novus Biologicals, Little, Colorado USA) overnight at 4°C. The sections were then incubated with horse radish peroxidase conjugated secondary antibody and visualized using diaminobenzidine (DAB).

### 2.11 Serum chemistry

These studies were carried out on blood obtained from the left ventricle at sacrifice. Assays of serum alanine aminotransferase (ALT), sodium, and urea nitrogen were performed by the clinical chemistry laboratory of the Iowa City VA Medical Center by automated methods. ALT was done by coupling the ALT dependent reaction between L‐argine and 2‐oxoglutarate to NADH oxidation by pyruvate and lactate dehydrogenase. Sodium was determined using an ion‐selective electrode. Urea nitrogen was determined by urease catalyzed NH_4_^+^ formation and coupling to NADH reduction by 2‐oxoglutarate and glutamate dehydrogenase.

### 2.12 Statistics

Data were analyzed by 2‐factor anova or 2‐tailed, unpaired *t* test as indicated in the figures and legends. Significance required *P* at least <.05.

## 3 RESULTS

### 3.1 Body mass and composition

**Figure 2:**
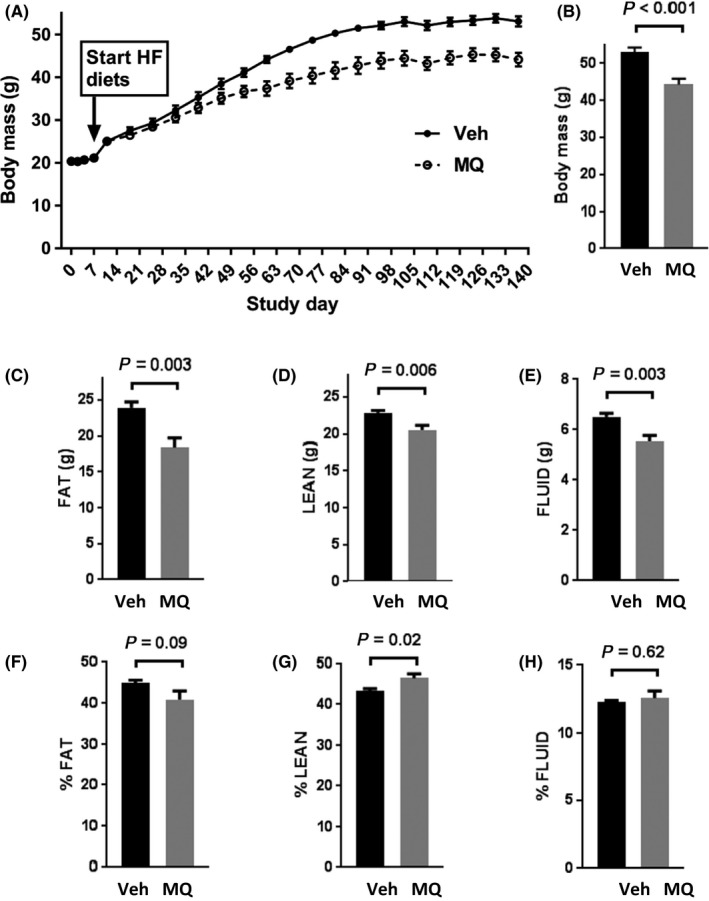
Body mass and composition. (A) Change in body mass over time in high fat (HF)‐fed, vehicle (water plus cyclodextrin) (Veh)‐treated mice and HF‐fed mitoquinone (MQ)‐treated (complexed with CD) mice. (B) Body mass at day 137 in the Veh and MQ groups. (C‐E) Body composition expressed in grams of fat, lean, and fluid mass, respectively, determined by NMR. (F‐H) Body composition expressed as % fat, % lean or % fluid mass determined by NMR. Data represent mean ± SE. n = 9 for Veh, n = 10 for MQ mice. *P* values were determined by 2‐tailed, unpaired *t* test

As expected, body weight increased markedly after 137 days of HF feeding in both the mitoQ‐ and vehicle‐treated groups (Figure 2A and B). However, the mean body weight at day 137 in the mitoQ group was 17% less than that in the vehicle‐treated mice and the change over baseline was reduced by 27%. Body composition was examined by NMR spectroscopy near study end (Figure 2C‐H). MitoQ reduced fat, lean, and fluid mass in about the same proportion (figure 2C‐E). At day 137, percent lean mass was significantly increased in the mitoQ group while percent fat was nonsignificantly (*P* = .09) decreased (Figure 2F and G). There was no difference in percent fluid (Figure 2H).

### 3.2 Food and water intake

**Figure 3:**
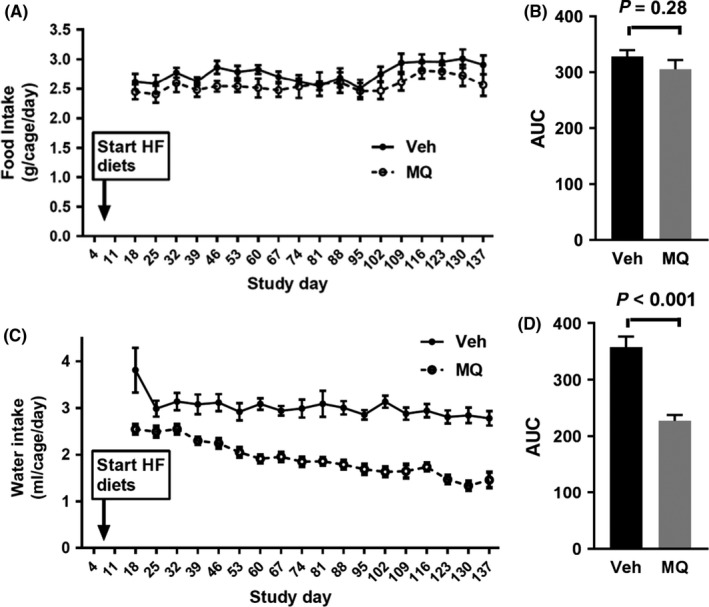
Food and water intake indicated by group. (A) Food intake (average per cage) measured on study days indicated over 7 d periods, beginning from days 11‐18 and depicted over intervals since prior x‐axis value. (B) Relative area under curve (AUC) for the data in (A). (C) Water intake (average per cage) measured on study days as indicated in (A) for food intake. (D) Relative area under curve (AUC) for the data in (C). Data represent mean ± SE. n = 4 for Veh group, n = 5 for MQ group. *P* values were determined by 2‐tailed, unpaired *t* test

Food and water intake were monitored after the onset of HF feeding (Figure 3). As shown in Figure 3A and B, food intake was mildly but not significantly reduced in the mitoQ‐ compared to vehicle‐treated mice. Consistent with past reports by ourselves and others,^7^, ^8^, ^9^ water intake by the mitoQ‐treated mice was reduced (Figure 3C and D).

### 3.3 Whole body energetics

**Figure 4:**
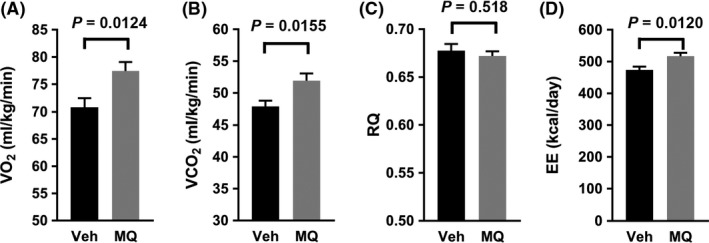
Whole body bioenergetic effects of mitoQ. (A) VO_2_. (B) VCO_2_. (C) Respiratory Quotient (RQ). (D) Energy expenditure (EE). Data represent mean ± SE. n = 8 for Veh, n = 10 for MQ mice. *P* values were determined by 2‐tailed, unpaired *t* test

Both resting whole body VO_2_ and carbon dioxide production (VCO_2_) were greater in the mitoQ‐ compared to vehicle‐treated mice (Figure 4A and B) while the respiratory quotient (RQ or VCO2/VO2) did not differ (Figure 4C). Energy expenditure estimated by the modified Weir equation^12^ was greater in the mitoQ‐treated mice (Figure 4D).

### 3.4 Insulin secretion and sensitivity

**Figure 5:**
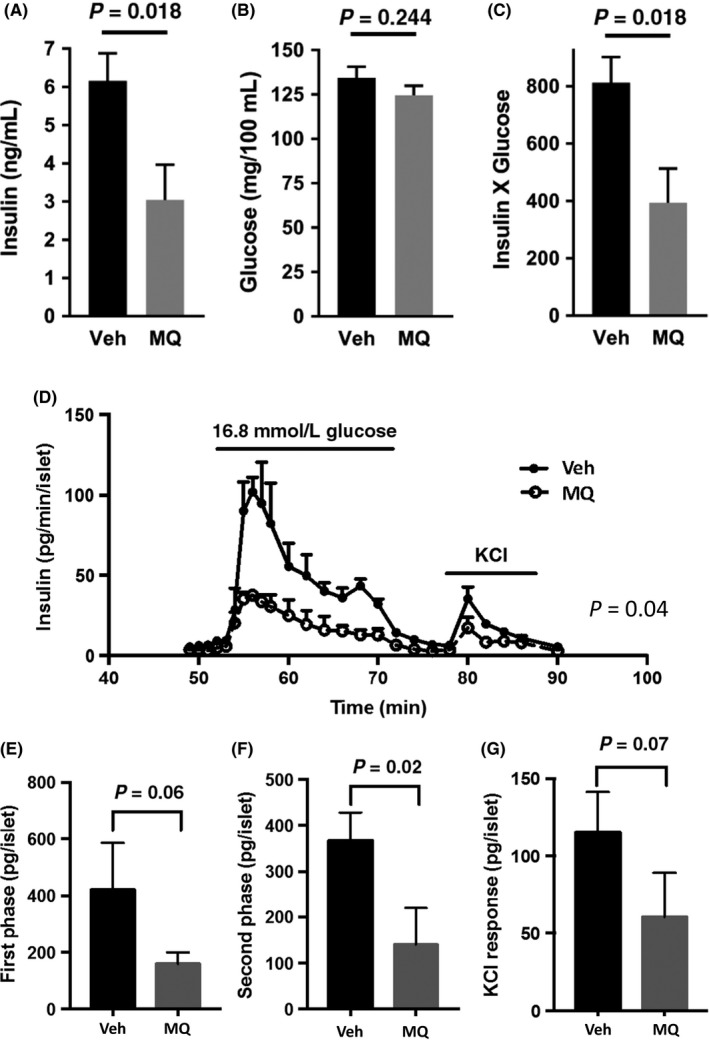
Whole body plasma insulin and glucose and glucose stimulated insulin secretion from isolated perifused islets. (A and B) Plasma insulin and glucose after overnight fast. (C) Insulin x glucose product. Data in (A‐C) represent mean ± SE. n = 9 for Veh, n = 10 for mitoQ (MQ) mice. (D) Insulin secretion in perfusate obtained from islets isolated from mice treated with Veh or MQ. (E‐G) Area under the curves of (D) for first phase insulin secretion (E, time 55‐60), second phase (F, time 62‐72), and KCL induced (G, time 80‐86). Data in (D‐G) represent mean ± SE. n = 3 per group. *P* values were determined by 2‐tailed, unpaired *t* test except for (D) in which a 2 way anova test was performed using the factors, time (as a repeated measure) and treatment group. The *P* value shown in (D) represents the treatment effect. As expected, both time and interaction were significant (*P* < .0001) consistent with the concept that the effet of mitoQ is time dependent

Fasting insulin concentrations were reduced by about 50% in the mitoQ‐ vs vehicle‐treated mice while glucose did not differ significantly (Figure 5A and B). Hence, insulin sensitivity was proportionally improved when calculated as the insulin x glucose product analogous to the human homeostasis (HOMA) model (Figure 5C).

Insulin secretion (expressed per islet) was measured in islets isolated at sacrifice and perifused in vitro (Figure 5D). These data revealed a marked decrease in insulin release from islets of the mitoQ‐treated mice when tested by 2 way anova. When area under the curves for phasic insulin secretion were compared (Figure 5E‐G), islets of the mitoQ‐treated mice showed a significant reduction in the second phase of glucose‐stimulated insulin secretion and a trend toward reduction in first phase and KCl responses.

### 3.5 Islet size

**Figure 6:**
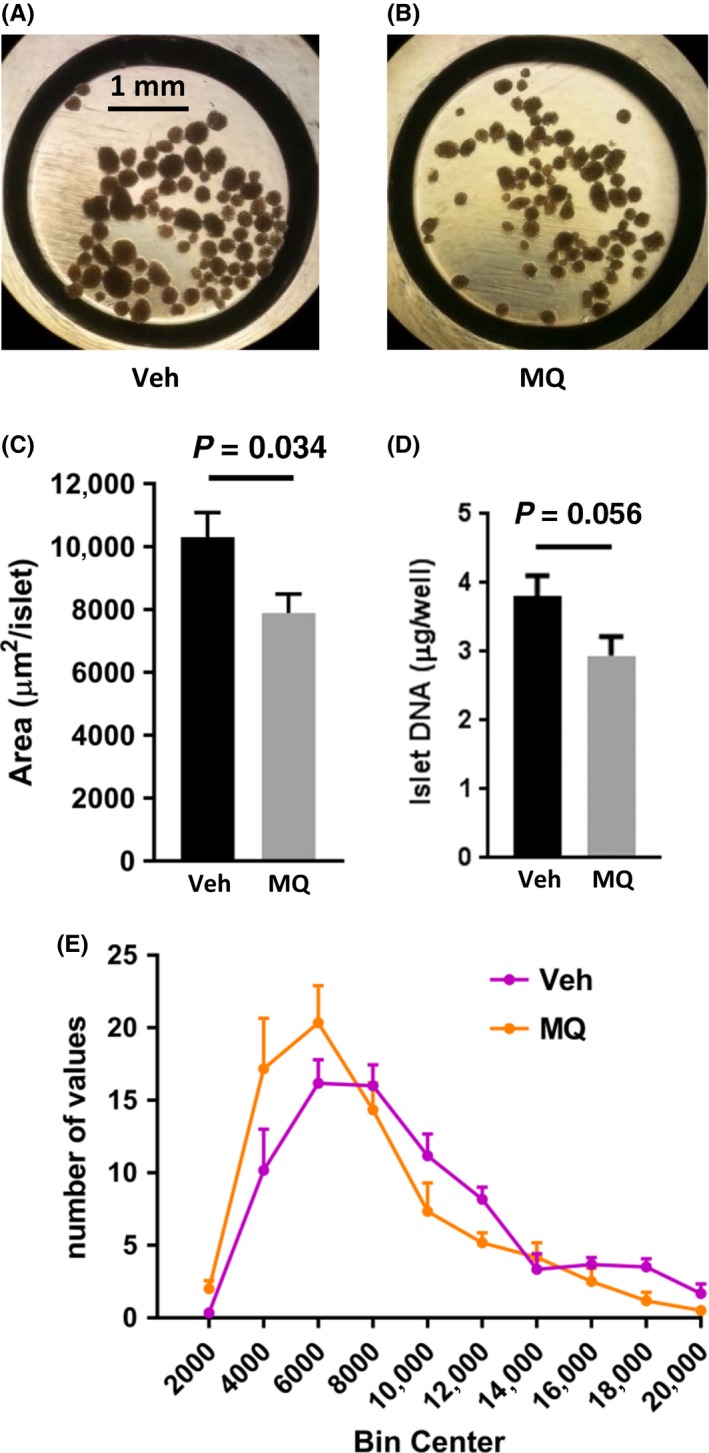
Islet size and DNA content. (A and B) Representative images of islets within wells of the islet plates used for Seahorse respirometry. Islets were obtained from a vehicle (Veh)‐ or mitoQ (MQ)‐treated mouse (C) Islet area (mean ± SE, n = 6 per group. (D) Islet DNA content measured in Seahorse wells after respirometry for islets of (C). (E) Histogram depicting the distribution of islet area for the Veh and MQ groups of (C). *P* values for (C and D) were determined by 2‐tailed, unpaired *t* test

Representative images of isolated islets within individual wells of the islet respirometer plates are shown in Figure 6A and B. Image analysis revealed that the average islet area of the mitoQ‐treated mice was significantly reduced (Figure 6C) while DNA content (Figure 6D) was likewise decreased (*P* = .056) indicating that mitoQ‐treated islets are smaller partly due to a smaller number of cells per islet. Islet size distributions for all islets of all mice in each group are depicted in Figure 6E.

### 3.6 Islet respiration and mitochondrial function

**Figure 7:**
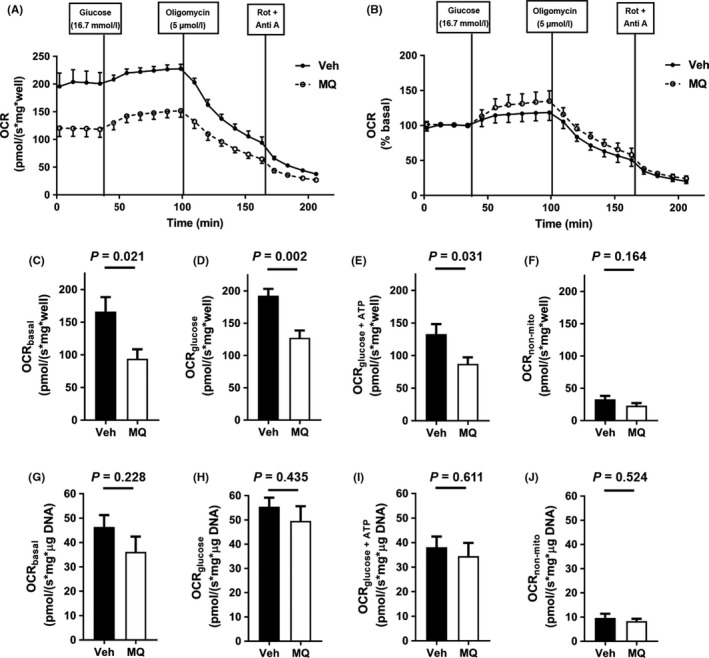
Respirometry data for islets obtained from vehicle (Veh)‐ or mitoQ MQ)‐treated mice. (A) Oxygen consumption rate (OCR) measurements over time and after additions of glucose, oligomycin, or rotenone (Rot) plus antimycin A (Anti A). (B) Data of (A) expressed as percent basal OCR. (C‐F) OCR component analyses expressed per well as basal OCR (C), glucose‐stimulated‐OCR (D), OCR directed at ATP production (E) and nonmitochondrial OCR (F). (G‐J) Parameters measured in (C‐F), respectively, but normalized to DNA content. Data in all panels represent mean ± SE, n = 6 islet preparations for each group. *P* values were determined by 2‐tailed, unpaired *t* test

Mean OCR measurements over time, and as related to additions to the respiratory media, for islets isolated from mitoQ‐treated or vehicle‐treated mice are plotted in Figure 7A and B. For all mice (six per group), respiratory component analyses showed that basal OCR, glucose‐stimulated‐OCR, and OCR directed at ATP production were all significantly reduced for islets isolated from mitoQ‐ vs vehicle‐treated mice (Figure 7C and E) while nonmitochondrial OCR did not differ (Figure 7F). When the same parameters were expressed per DNA content of each well rather than per islet, the difference between groups lost significance (Figure 7G‐J).

### 3.7 Renal and hepatic function and plasma sodium

Plasma analytes and liver fat content measured at sacrifice are depicted in Table 1. ALT, a well‐recognized marker of liver function, was markedly improved by mitoQ treatment while liver fat content was reduced by approximately one‐third. Plasma sodium was slightly increased. Urea nitrogen did not differ between mitoQ‐treated and vehicle‐treated mice. Creatinine concentrations (a standard marker of renal function in humans) were not measured as it is known to be low in mice compared to humans and in past studies, our institution's laboratory was not able to accurately detect this analyte in mice.^8^, ^13^ Note also that urea nitrogen, another well‐known marker of renal function in humans, is a better marker for hydration than creatinine and is elevated more than creatinine in dehydration.^14^

**Table 1:** Effect of Mito Q (MQ) compared to vehicle (Veh) treatment on the concentration of analytes in plasma obtained at sacrifice and fat content in liver (mean ± SE). *P* values were determined by 2‐tailed, unpaired *t* test, n = 9 for Veh, n = 10 for MQ

Analyte	Veh	MQ	*P* value
Alanine aminotransferase (U/L)	223 ± 29	69 ± 17	.0002
Liver fat (mg/g wet weight)	217 ± 8	148 ± 19	.0046
Urea nitrogen (mg/dL)	24.6 ± 1.2	26.7 ± 2.5	.47
Sodium (mEq/L)	153.2 ± 0.4	155.6 ± 0.5	.002

### 3.8 Islet hydroperoxide content

**Figure 8:**
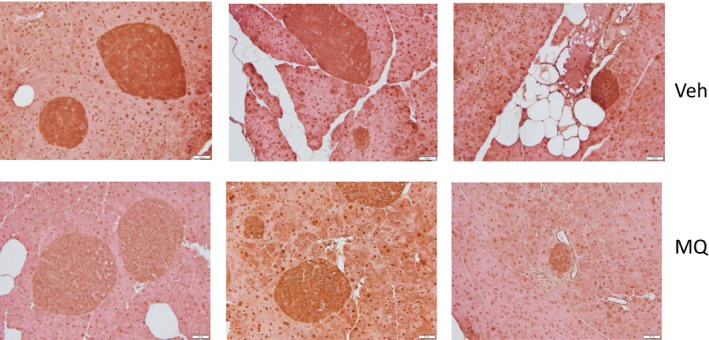
Islet hydroperoxide staining. Representative pancreatic sections obtained from three different vehicle‐treated (Veh) or mitoQ‐treated (MQ) mice. Immunohistochemical staining was performed using antibody directed at malondialdehyde (MDA) as a marker of lipid peroxides

Although not quantified, staining for islet MDA (Figure 8) was less intense in random sections of pancreatic tissue from MQ‐treated mice compared to Veh‐treated (3 mice per group).

## 4 DISCUSSION

Obesity‐induced insulin resistance and susceptibility to diabetes are worldwide problems often resistant to efforts at modifying lifestyle.^15^ Because available drug therapy is, at best, only partially effective and associated with side effects, continued efforts are underway to develop new pharmacologic approaches. In recent studies, we^7^, ^8^ and others^16^ have found that mitoQ administered to obesity prone mice mitigates weight gain acting by decreasing appetite and/or increasing energy expenditure, the latter likely due to mild respiratory uncoupling.

Despite what is known about mitoQ on body energetics, little is known about the effect of mitoQ on insulin secretion. Lim, et al^17^ exposed cultured insulin producing RINm5F and HIT‐T15 insulinoma derived β‐cells to glucotoxic and lipotoxic conditions in the presence of absence of 100 nmol/L mitoQ. They reported that mitoQ protected these cells from oxidative damage and loss of glucose‐induced insulin secretion. However, that study did not address islet β‐cell function as affected by mitoQ administration to live animals. Here, we report novel data regarding the effect of the mitochondrial‐targeted compound, mitoQ on pancreatic islet size and function when administered to obesity‐prone HF‐fed mice. HF feeding to these mice is well‐known to induce substantial weight gain, insulin resistance, and compensatory hyperplasia of pancreatic islets.^18^, ^19^

NMR body composition analyses of the mitoQ‐ compared to vehicle‐treated mice revealed reduced mass of all measured components (fat, lean, and fluid) (Figure 2C‐E). However, there was a greater reduction in fat mass associated with a significant increase in percent lean mass and near significant decrease in percent fat (figure 2F and G). These changes could have resulted from decreased caloric intake or increased energy expenditure, both of which were observed (Figures 3A and B and 4D); although, only energy expenditure was significantly different. As expected based on the above changes in body composition, mitoQ‐treated mice exhibited greater insulin sensitivity as indicated by the insulin × glucose product and considerably lower fasting insulin despite little difference in glucose (Figure 5A‐C). Although the reduction in body weight and body fat may seem small in comparison to the decrease in insulinemia and improvement in insulin resistance, this is not surprising since it is well‐known that small amounts of weight loss, as little as 5%, can substantially improve glycemia and insulin sensitivity in humans.^20^

Our results regarding insulin secretion and respiration by isolated islets were consistent with our observations regarding islet size as well as whole‐body energetics and insulin sensitivity. Upon isolation and visual inspection of our islet preparations under the light microscope, it was apparent that the islets of our mitoQ‐treated mice were smaller in size. This was confirmed by image analysis as shown in Figure 6. This was also consistent with our observation that DNA content was reduced, although at a borderline *P*‐value of .056 by 2‐tailed analysis. Glucose‐induced insulin secretion was reduced in islets from mitoQ‐ compared to vehicle‐treated mice (Figure 7D‐G). Moreover, islet VO_2_ under basal and glucose‐stimulated conditions as well as VO_2_ directed at ATP production by the mitoQ‐treated islets was reduced when the OCR values were expressed per islet (Figure 7C‐E). We believe that this was largely consequent to the decrease in islet size since the data did not significantly differ when OCR was expressed per islet DNA (Figure 7G‐I). Hence, mitoQ treatment of live mice subject to HF‐induced obesity results in changes at the islet level consistent with a reduced demand for insulin. In other words, mitoQ treatment resulted in a more favorable energetic state requiring less insulin and at least mitigating the demand for obesity associated islet hypertrophy.

Although the decrease in body fat and improved insulin sensitivity explains much of the decrease in insulin need by mitoQ treated mice, other factors may have played a role. MitoQ markedly improved liver function based on the ~3‐fold reduction in plasma ALT, a commonly used clinical marker of hepatic dysfunction. In past work, we showed that mitoQ decreased liver hydroperoxides by about 50% in HF‐fed mice.^8^ In this study, mitoQ treatment reduced liver fat content by about 33% (Table 1). Liver steatorrhea is strongly associated with insulin resistance,^21^ so the effect of mitoQ to improve liver function may contribute to the decrease in demand for insulin in our HF‐fed model. It is known that insulin resistant liver produces humoral and neurological signals to increase beta cell mass.^22^, ^23^ Thus, the prevention of fatty liver and liver injury by mitoQ might have reduced hepatic signals that drive a compensatory increase in beta cell mass. Of course, a caveat to this logic is that it remains debatable whether liver steatorrhea causes insulin resistance or whether insulin resistance causes steatorrhea.

β‐cell lipid deposition and oxidized lipids are known to impair insulin release.^24^ In this regard, our immunohistochemical imaging suggests that mitoQ mitigated mitochondrial oxidative damage as evidenced by the decrease in islet hydroperoxide content (Figure 8). So, by virtue of its known action to inhibit lipid peroxidation,^25^ mitoQ may have improved islet capacity for insulin secretion. However, the hepatic and systemic effects of mitoQ presumably decreased insulin demand to the point that we could not directly observe a beneficial effect of mitoQ on insulin secretion per se. In addition, we can at least speculate that mitoQ improved the efficiency of islet metabolism contributing to the reduced need for islet VO_2_.

Our current results are largely consistent with our past studies of the action of mitoQ on HF‐fed mice. In both this study and in past reports^7^, ^8^ we found that mitoQ improved energy expenditure (based on gas respiratory exchange) and decreased food intake. However, in our past reports, unlike the present, the effect on whole body respiration was short of significance. Likewise, our past study found a significant decrease in food intake, whereas in the present study the decrease in intake was short of significance. These differences may have been due to variation in the results. Another difference is that in the current study mitoQ significantly reduced hepatic fat content, whereas we did not observe this change in our above mentioned prior report. Differences between our present and prior report include the exact time of treatment and dosing of mitoQ, although both studies eventually increased mitoQ in the drinking water to 500 μmol/L. Furthermore, our past study used bromide as the mitoQ anion and did not include CD. Here we used the mesylate salt with CD, possibly a more favorable method.^9^, ^16^, ^26^ So, it is possible that in our current study, we were simply more efficient in administering mito Q. In both our current and past studies, we found that mitoQ markedly and significantly decreased ALT, although more so in the present report.

As in prior reports from our laboratory and others,^7^, ^8^, ^9^, ^16^ water intake was reduced in the mitoQ‐treated mice compared to vehicle‐treated. As in our past work, this was not accompanied by a difference in percent body fluid or urea nitrogen. Unlike our past work where plasma sodium did not significantly differ, we did observe a slight increase in sodium of 2.4 mEq/L, a difference that, at least for human physiology, would be considered of minimal, if any, clinical importance.

There are limitations to our study. We examined only HF‐fed mice. In a past study,^8^ which did not include measures of pancreatic islet function, we treated normal mice with mitoQ and observed no differences in body mass or body composition (fat, lean, and fluid mass) at least suggesting no change in insulin sensitivity. Although we observed that mitoQ mitigated the islet hypertrophy that typically occurs in obesity,^27^ we do not know whether the resultant islet size differs from what would be expected for nonobese mice fed normal chow and followed up for the same time. However, we previously noted that islet area of adult C57BL/6J is typically below 5000 μm^2^ supporting that islets in our study are enlarged.^19^ A further limitation is that we show the effects of mitoQ on islet function in a preventative fashion which does not actually mimic treatment of already existing obesity.

In summary, mitoQ treatment mitigated islet hypertrophy and reduced both islet VO_2_ and insulin secretion. These effects were likely due to more favorable energetics and reduced body mass implying that mitoQ improved insulin sensitivity and reduced the demand for insulin release. The decreased demand may also have resulted from improved liver function.

## AUTHOR CONTRIBUTIONS

Participated in research design: Imai, Fink, Sivitz; Conducted experiments: Imai, Fink, Promes; Contributed new reagents or analytic tools: Kulkarni, Kerns; Performed data analysis: Imai, Fink, Sivitz; Wrote or contributed to the writing of the manuscript: Imai, Fink, Sivitz.
